# Comparison between tropical legumes and natural grasses in improving tropical rainforest soil health: a case study in guava (*Psidium Guajava* L.) orchards

**DOI:** 10.1186/s12870-025-06395-z

**Published:** 2025-03-25

**Authors:** Xiaoping Zang, Kai Li, Tianyan Yun, Afaf Abdullah Rashed, Dalia Mohammad Melebari, Zheli Ding, Hanan Elsayed Osman, Mamdouh A. Eissa, Yingdui He, Tao Jing, Lixia Wang, Yongxia Liu, Jianghui Xie, Weihong Ma, Changbin Wei

**Affiliations:** 1 National Key Laboratory for Tropical Crop Breeding, Institute of Tropical Bioscience and Biotechnology & Sanya Research Institute, Chinese Academy of Tropical Agricultural Sciences, Haikou 571101/Sanya 572024, China; 2 Biology Department Faculty of Science Umm Al-Qura University, Makkah, Saudi Arabia; 3 Botany and Microbiology Department, Faculty of Science, Al-Azhar University, Cairo, Egypt; 4 Department of Soils and Water, Faculty of Agriculture, Assiut University, Assiut, 71526 Egypt; 5 Tropical Crops Genetic Resources Institute, Chinese Academy of Agricultural Sciences, Haikou, 571101 China; 6 Key Laboratory of Tropical Fruit Biology, Ministry of Agriculture & Rural Affairs, South Subtropical Crops Rsearch Institute, Chinese Academy of Agricultural Sciences, Zhanjiang, Guangdong 524091 China

**Keywords:** Orchard grass, Latosol, Soil microorganisms, Richness and evenness, Β- diversity

## Abstract

Tropical rainforest soils, or latosols, are distinguished by their low pH and low fertility. In orchards, co-cultivating grass has become popular as a way to improve soil quality and boost fruit production. Nevertheless, insufficient information is currently available about the response of soil microbial communities in tropical rainforest orchards to grass co-cultivation. Therefore, the present research investigates the effect of grass cultivation on the soil properties and microbial diversity of guava (*Psidium guajava* L. cv Pearl) latosol orchards. Two varieties of the tropical legume grass *Stylosanthes guianensis*, i.e., Reyan No. 2 and Ubon, were studied, besides the control (CK), which is without any grass, and the natural grasses treatment (N). The study contained four treatments, i.e., *S. guianensis* cv. Reyan No. 2, *S. guianensis* cv. Ubon, CK, and N. Soil samples from the top layer (0–20 cm) and subsoil layer (20–40 cm) were collected to follow the changes in soil microbial biodiversity based on 16 S rDNA analysis. A total of 17,231 kinds of OTUs (Operational Taxonomic Units) were obtained, including 17,165 kinds of bacteria and 66 kinds of *Archaea*. *S. guianensis* cv. The Ubon variety, natural grasses, and CK treatments significantly increased the soil microbial richness and evenness in the topsoil layer compared to Reyan No. 2 variety. The β-diversity of soil microbial community was significantly reduced in the natural grasses and Ubon variety treatments at the topsoil layer compared to CK treatment. In the subsoil layer, natural grasses, Reyan No. 2, and Ubon treatments significantly increased the soil microbial community based on β-diversity. The presence of natural grasses caused 49% and 42% increases in the SOC in the top and subsoil layers, respectively, as well as remarkable increases in the available and total soil nitrogen. The grass intercropping enhanced the levels of soil carbon and nitrogen and altered the nature of the soil’s microbial community. The diversity of soil microorganisms in the subsoil layer is significantly altered by the shallow root systems of tropical legume and natural grasses, which have most of their roots concentrated in the top soil layer. Overall, growing grass in tropical orchards benefits the latosolic soil microorganisms, which has enhanced the theoretical underpinnings for using grass to improve the soil quality in latosols orchards.

**Supplementary Information:**

The online version contains supplementary material available at 10.1186/s12870-025-06395-z.

## Introduction

The cultivation of grass in orchard fields is a method that was created in the middle of the 20th century as a sustainable management model and offers several positive ecological and economic benefits [1]. Grass cultivation technique has the characteristics of being compatible with nature, comprehensively improving the production capacity of orchards [2, 3]. Clean tillage is still a common management technique even though growing grass in orchards is a useful tool for sustainable development [4]. Fruit orchards are an essential part of agriculture and have expanded by almost 22% in the last ten years to meet the growing demand for fruit consumption [5, 6]. Improving orchard management is urgent in order to facilitate carbon sequestration and improve soil health [7]. Maintaining grass cover in orchards has been demonstrated to raise the SOC content, promote healthy soil, and eventually increase fruit production [1, 3, 8]. Growing grass in orchard could increase porosity, improve soil structure, and enhance nutrients availability [1]. Once grass is grown in an orchard, the number of probiotics, enzyme activity, and soil microbes can all rise considerably [9–11]. Soil health response of tropical and subtropical orchards to the grass cultivation is unknown and requires more experimental scientific research to discover the best types suitable for this unique type of soil.

Soil microorganisms influence soil ecosystems and affect a large number of vital ecosystem processes, including soil energy flow, element cycling, mineralization and decomposition of SOC, and the supply of essential nutrients for crop growth [12, 13]. At least 25% of all biodiversity on Earth is found in soil microbiome [14]. There are tens of millions of species of microeukaryotes, viruses, bacteria, and fungus in the world; just a few hundred thousand of them have been fully studied [15]. In earth’s ecosystems, their biomass, diversity, and activity serve as sensitive markers of soil productivity, quality, and sustainability [12, 13]. Changes in plant biomass and species are expected to affect the activity and abundance of microbial functional groups since different plants have distinct physiological traits [1, 11]. Plants create distinct microbial communities in soils at varying stages of degradation by controlling microorganisms through litter and root exudates [11, 16]. Studies have shown that interplanting grass has a significant effect on the structure and function of soil bacteria, including community diversity and carbon metabolism-related activities [2, 17]. Nonetheless, the different types of grass interplanting have distinct effects on the composition and capabilities of the soil bacterial populations [1, 10]. Thus, it is crucial to comprehend the effects of sowing grass in a guava orchard on soil nutrients and the variety of the bacterial population in order to manage tropical latosol orchards.

The tropical soils formed under hot and wet tropical conditions causing intense leaching, depleting essential cations such as calcium, magnesium, and potassium, while leaving iron oxides as the dominant compounds [19, 20]. This process has contributed to extreme acidity and low organic carbon levels, posing significant challenges for tropical soil management and fertility [19, 20]. The effect of grass cultivation on most of the previous studies conducted using ryegrass, *Leymus chinensis*, white clover, and animal forage plants under alkaline soils of non-orchard field [11, 18]. *Stylosanthes gianensis*, as an excellent perennial leguminous forage, is widely cultivated in tropical regions in China [19, 20]. The impact of *S. gianensis* will have on soil microorganisms in tropical latosol soil orchards has not been reported. We hypothesized that different types of grass cover changed the compositions of the soil microbial communities in latosol orchards; furthermore, these changes may have been linked to soil characteristics. Consequently, the goal of this research is to explore the soil microbial community using the 16 S rDNA high-throughput sequencing technique to understand the significance of *S. gianensis* and the natural tropical grasses on the soil microorganisms. Moreover, the study aims to provide theoretical guidance for the use of grass cultivation on soil improvement and sustainability of latosol orchards.

## Materials and methods

### Site and design of the experiment

**Fig. 1:**
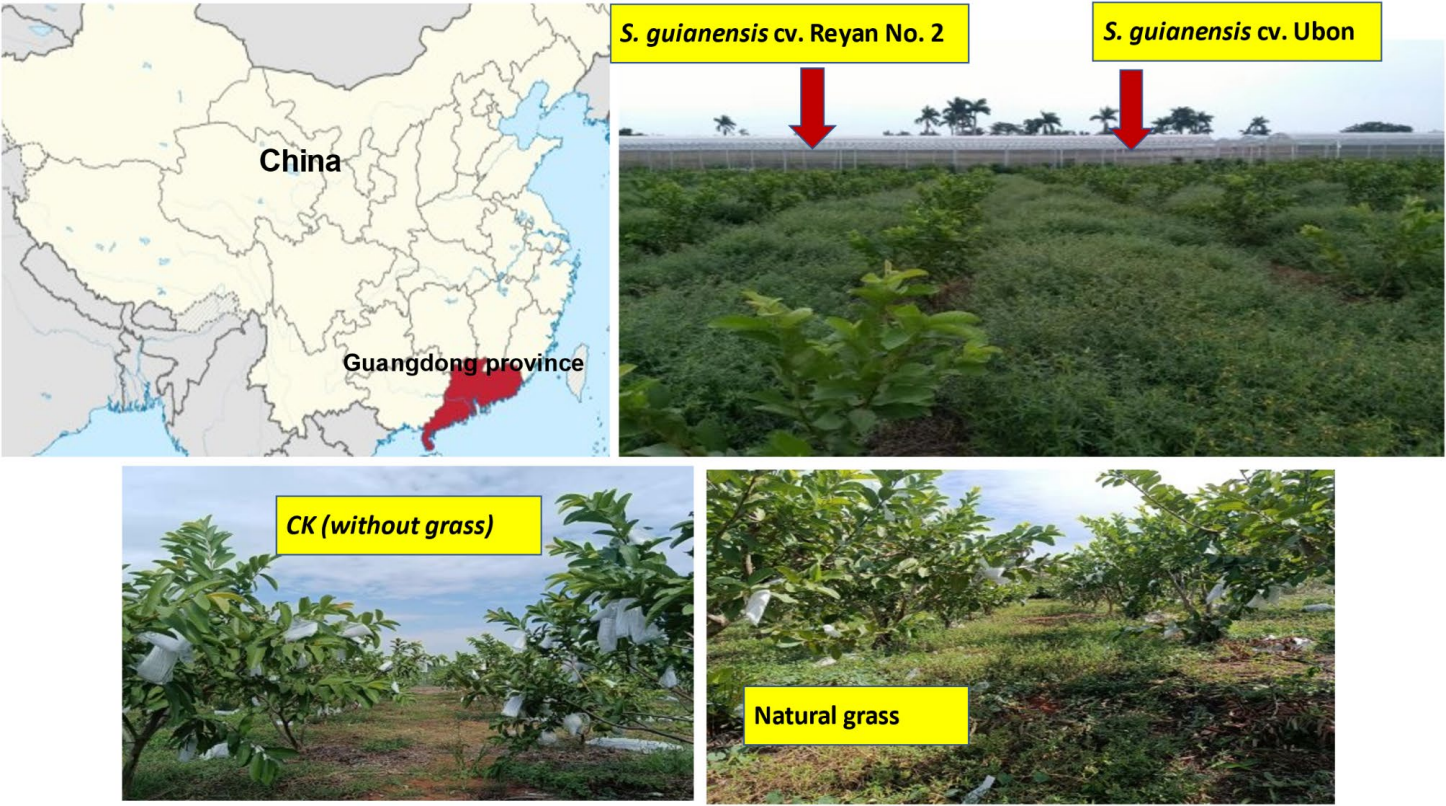
Site and treatment

The experimental site is situated in a guava orchard within the experimental base’s orchard (110° 28 E, 21° 16 N) of the Institute of South Subtropical Crop Research in Zhanjiang, Guangdong, China. The Zhanjiang region is subtropical, with very mild winters and a hot, humid, and rainy summer with an average annual precipitation of 1795 mm. Supplementary Figure S1 shows the main climate conditions. The tested latosolic soil has a pH value of 6.74 in 0.01 M CaCl_2_ and contains 11.05 and 1.1 g kg^− 1^, respectively, of organic carbon and total nitrogen. The concentrations of the available nitrogen, phosphorus, and potassium are 100, 9.20, and 200 mg kg^− 1^, respectively. The seedlings of guava (*Psidium guajava* L. cv Pearl) were cultivated in April 2016 at 2.0 m × 3.0 m spacing. Two varieties of *S. guianensis*, i.e., Reyan No.2 and Ubon, besides the natural grass, were cultivated at the same time in the experimental field. The natural grass treatment is a natural weed in guava fields in the Zhanjiang region and consists mainly of *Eleusine indica* (L.) Gaertn, and *Echinochloa crusgalli* (L.) Beauv. The source of all the tested plants used in this study is the Institute of Tropical Crops Genetic Resources, Chinese Academy of Tropical Agricultural Sciences, Hainan, China. *S. guianensis* cv. Reyan No. 2 and *S. guianensis* were cultivated at a seeding dose of 10 kg h^− 1^ [21]. Compound fertilizer was applied by drip irrigation four times a year, and the fertilization was located on the tree disk Fig 1.

The tested treatments were *S. guianensis* cv. Reyan No. 2 (represented by E), *S. guianensis* cv. Ubon (represented by F), natural grass (represented by N), and clean tillage treatment (represented by CK). Each treatment consisted of 6 lines, and each line represented an experimental plot of 2.0 m × 15 m. A randomized complete block design (RCBD) with five replicates was used to set up the experiment. Twice a year, the grasses in the E, F, and N treatments were mowed; the clipped grasses were then dispersed along the guava rows. Monthly weeding was done in the control treatment (CK) to maintain a clean cultivation throughout time. The soil samples were collected in October 2018. Soil samples were randomly collected from the experimental plot at two different depths (between 0 and 20 and between 20 and 40 cm). Each sample consisted of three sampling points. Once the samples were gathered, they were promptly preserved by freezing them in liquid nitrogen. Subsequently, the frozen samples were sent to the laboratory for further investigation.

### Experiment procedure

#### Basic soil analysis

The soil pH, organic matter, total and available N, P, and K were determined according to the method of Lu [22].

#### The extraction of DNA and amplification of PCR

Following the manufacturer’s instructions, microbial DNA was extracted from the soil samples using the soil DNA Kit (FastDNA^®^ Spin Kit for Soil MP bio U.S.). The Eukaryotic ribosomal RNA gene’s 16 S rDNA V3-V4 region was amplified by PCR using primers 341 F, which is CCTACGGGNGGCWGCAG, and 806R, which is GGACTACHVGGGTATCTAAT. The PCR was conducted for two minutes at 95 °C, followed by 27 cycles at 98 °C for 10 s, 62 °C for 30 s, and 68 °C for 30 s, and a final extension at 68 °C for 10 min. The barcode is an eight-base sequence specific to each sample. PCR experiments were carried out in triplicate using a 50 µL mixture that included 1 µL of KOD Polymerase, 5 µL of 2.5 mM dNTPs, 1.5 µL of each primer (5 µM), and 100 mg of template DNA.

#### Illumina Hiseq2500 sequencing

Following the manufacturer’s recommendations, amplicons were extracted from 2% agarose gels, purified using the AxyPrep DNA Gel Extraction Kit (Axygen Biosciences, Union City, CA, U.S.), and quantified using QuantiFluor -ST (Promega, U.S.). The equimolar pooling of purified amplicons was followed by paired-end sequencing (2 × 250) on an Illumina platform using conventional methods.

### Bioinformatics analysis

#### Quality assurance and assembly reading

After further filtering raw reads, paired end clean reads were merged as raw tags using FLSAH [23] (v1.2.11) with a minimum overlap of 10 bp and mismatch error rates of 2% in order to get high quality clean reads. The QIIME (V1.9.1) pipeline [24]was used to filter noisy raw tag sequences under certain filtering parameters [25] in order to produce high-quality clean tags. In order to perform reference-based chimera verification using the UCHIME algorithm(http://www.drive5.com/usearch/manual/uchime_algo.html), clean tags were searched against the reference database (http://drive5.com/uchime/uchime_download.html). After removing all chimeric tags, effective tags were retrieved for additional investigation.

#### OTU cluster

The UPARSE pipeline was utilized to cluster the effective tags into operational taxonomic units (OTUs) with a minimum of 97% similarity [26]. Each cluster’s repulsive sequence was chosen based on the tag sequence with the highest abundance. Venn analysis was done in R to find common and unique OTUs between groups.

#### Classification of taxonomies

RDP classifier (Version 2.2) based on SILVA database (https://www.arb-silva.de/) was used to classify the sample sequences into species using a naïve Bayesian model analysis [27, 28].

#### Alpha diversity analysis

Chao1, Simpson and all other alpha diversity indexes were calculated in QIIME. The statistical analysis of the Alpha index comparison between groups was conducted using R’s Welch’s t-test and Wilcoxon rank test. Both the Kruskal-Wallis H test and the Tukey’s HSD test in R were used to calculate the alpha index when comparing groups.

#### Analysis of beta diversity

QIIME developed a weighted and unweighted unfired distance matrix. The weighted unfired distances’ NMDS were computed and illustrated in R. The statistics tests of Welch’s t-test, Wilcoxon rank, Tukey’s HSD, Kruskal-Wallis H, Adonis were calculated using R.

#### Data analysis

STATISTIX 8.1 was utilized to do a one-way ANOVA and Tukey’s test at a 5% probability level in order to assess the significance of mean differences. Each table and figure’s data is presented as means ± standard deviation (SD, *n* = 5). The R library’s “factoextra” and “corrplot” functions were utilized to execute principal component analysis (PCA) and correlation matrix, respectively, using R software version 4.4.0.

## Results

### Basic soil characteristics

**Fig. 2:**
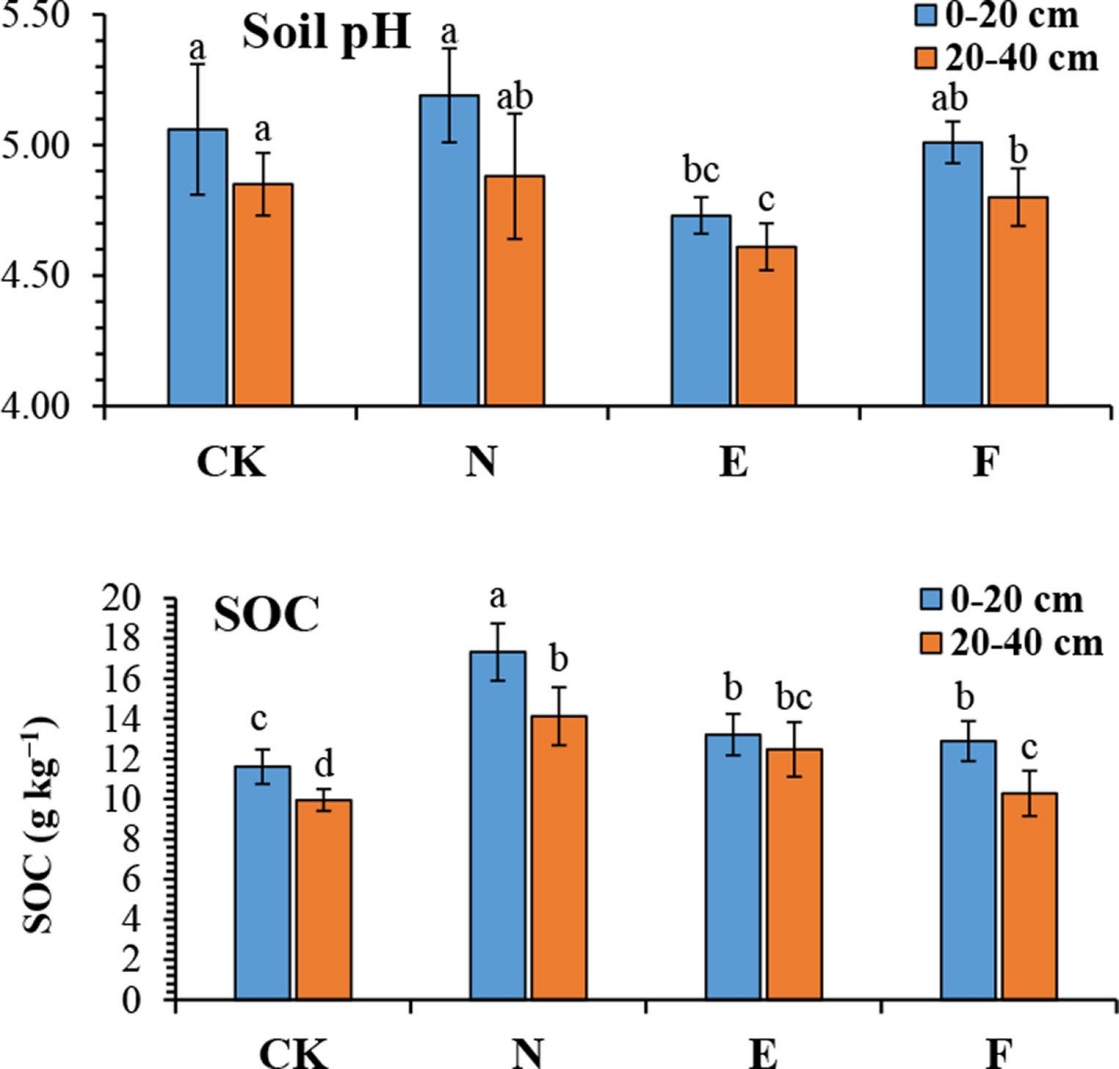
Effect of grass cultivation on soil pH and soil organic carbon (SOC). CK, N, E, and F: control without grass, natural grass, Reyan No. 2, and Ubon, respectivally. Values are means SD, *n* = 5. Means with the same letter indicate non-significant differences based on Tukey test (*p* < 0.05)

Table 1; Fig. 2 illustrate how the grass cultivation has altered the soil’s pH, essential plant nutrient content, and soil organic carbon (SOC). The natural grass treatment (N) had significantly (*p* < 0.05) greater total nitrogen and SOC than Reyan No. 2 (E), Ubon (F), and CK (without grass). In the top soil layer, the amount of available nitrogen in N treatment was significantly (*p* < 0.05) higher than that in Ubon and CK treatments. In the subsoil layer, the total nitrogen of N treatment was significantly (*p* < 0.05) higher than that of Ubon and CK, while the available nitrogen of N and E was significantly (*p* < 0.05) higher than that of CK. The natural grass caused 49 and 42% increases in the SOC in the topsoil and subsoil layers, respectively. The natural grass increased the total and available nitrogen by 56 and 34% in the topsoil layer, while these increases were 59 and 46% in the subsoil layer.

**Table 1:** Effect of plant type on nitrogen (N), phosphorus (P), and potassium (K) in surface (0–20 cm) and subsurafce soil (20–40 cm)

	0–20 cm				20–40 cm			
	CK	*N*	E	F	CK	*N*	E	F
Total nitrogen (g kg^−1^)	1.01±0.05bd	1.58±0.14a	1.13±0.02b	1.12±0.09bc	0.82 ±0.03bc	1.30 ±0.27a	1.06 ±0.08ac	0.90 ±0.09bc
Total P (mg/kg)	554.90±43.49a	587.61±109.22a	608.00±88.07a	638.42±92.56a	494.53 ±22.58a	457.60 ±40.10a	517.71 ±34.94a	480.15±123.90a
Total K (g kg^−1^)	2.99±0.09a	3.15±0.19a	2.78±0.06b	2.94±0.08ab	2.80 ±0.06a	2.87 ±0.15a	2.62 ±0.16a	2.78 ±0.12a
Available-N (mg kg^−1^)	121.94±12.09bc	162.92±16.52a	148.61±2.34ac	126.31±5.83bc	101.34 ±10.29bd	148.13 ±26.35ac	161.22 ±10.72a	108.37 ±18.81bc
Available -P (mg kg^−1^)	4.00±1.98a	9.98±6.77a	9.56±3.58a	17.53±9.13a	1.79 ±0.19a	3.84 ±2.24a	4.00 ±1.69a	7.73±4.38a
Available -K (mg kg^−1^)	189.74±40.93a	176.76±78.60a	229.35±25.34a	188.15±61.19a	115.88 ±21.08a	175.22 ±126.83a	194.04 ±46.18a	114.42 ±21.25a

CK, N, E, and F: control without grass, natural grass, Reyan No. 2, and Ubon. Means with the same letter indicate non-significant differences based on Tukey test (*p* < 0.05)

### Operational taxonomic units (OTUs) distribution

**Fig. 3:**
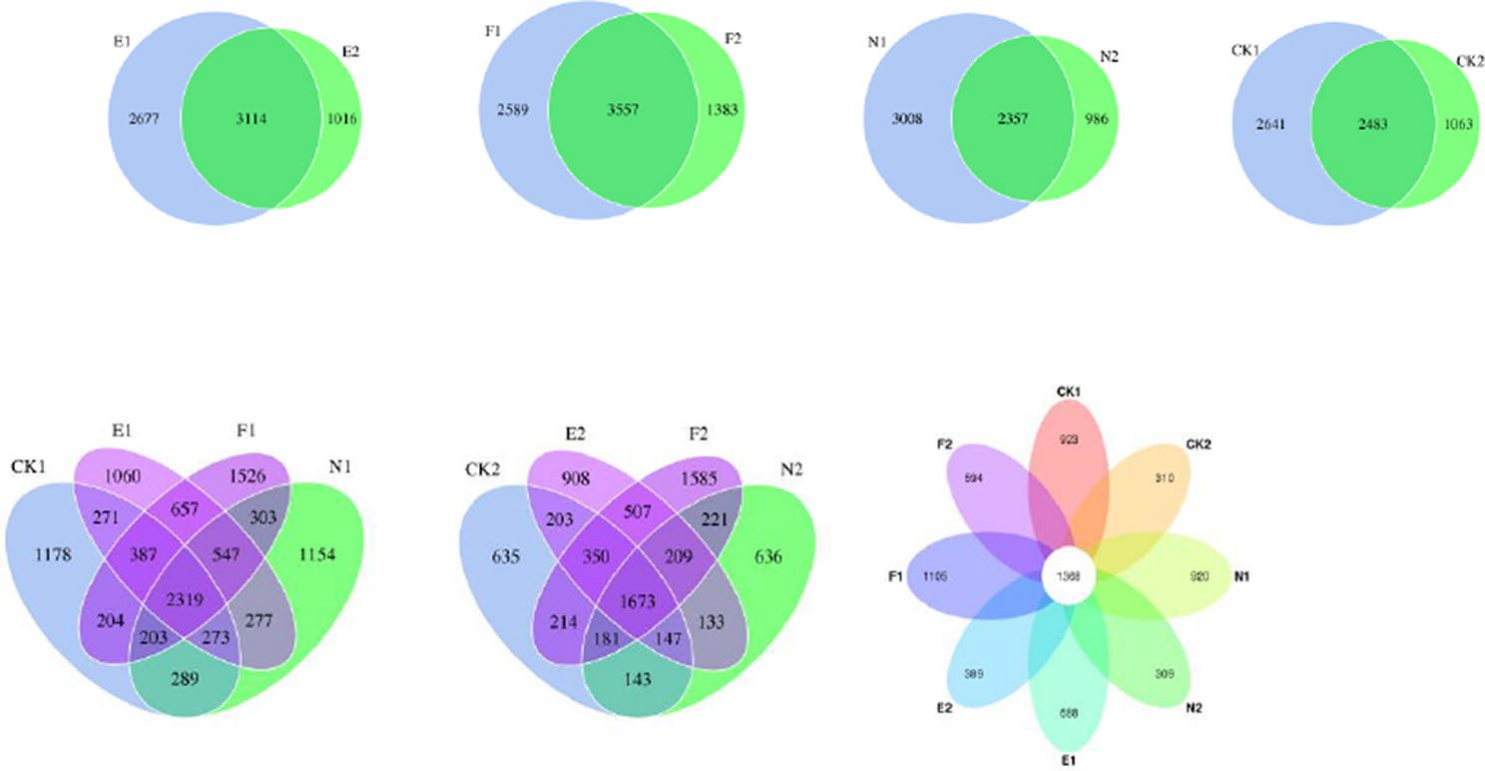
Venn diagrams of different treatments.1 and 2 refer to top and subsoil sample, respectively. CK, N, E, and F: control without grass, natural grass, Reyan No. 2, and Ubon

OTUs with 97% identity were formed from all of the soil sample effective tags. A total of 17,231 kinds of OTUs were obtained, including 17,165 kinds of bacteria and 66 kinds of Archaea (Supplementary Table S1). The venn diagram of each treatment is shown in Fig. 3, which shows that the total and proprietary OTUs number of soil microorganisms in the topsoil layer are higher than that in the subsoil layer. The number of OTUs shared by the topsoil and subsoil layers of E and F was higher than that of N and CK. In the topsoil layer, the order of proprietary OTUs number was in the descending order: F > CK > N > E, and in the subsoil layer, the order of proprietary of OTUs number was: F > E > N > CK.

### Community structure analysis of soil microorganism

**Fig. 4:**
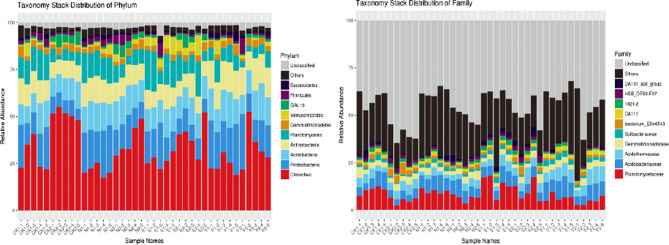
Taxonomic structure of the soil microbiota at the phylum and family level. 1 and 2 refer to top and subsoil sample, respectively. CK, N, E, and F: control without grass, natural grass, Reyan No. 2, and Ubon

According to the species annotation information of OTU, combined with the expression of OTU in the different soil samples, the expression of each sample at each classification level was calculated. Figure 4 provides the categorization data at the phylum and family levels. At the phylum level, the most common one was *Chloroflexi*, followed by *Proteobacteria*, *Acidobacteria*, *Actinobacteria*, and *Planctomycetes*. A percent of 2–3% of the tags was unclassified. In the E, F, and CK treatments, the topsoil layer had a lower relative abundance of *Chloroflexi* than the subsoil layer, and there was a significant difference in the relative abundance of CK between the two soil layers (*p* < 0.05). In the N treatment topsoil layer, the relative abundance of *Chloroflexi* was less than that of CK. However, the relative abundance of *Chloroflexi* in the subsoil layer in N treatment was significantly lower than that in the topsoil layer. The relative abundance of *Proteobacteria* in the topsoil layer of E, F, and CK treatments was higher than those of the subsoil samples, and the relative abundance in CK between the two soil depths was significantly different, but there was no significant (*p* > 0.05) difference between the two soil depths in N treatments. The relative abundance of *Proteobacteria* in the topsoil layer of E and F treatments was higher than that in CK and N (Supplementary Table S3).

At the family level, the dominant families are mainly *Planctomycetaceae*,* Acidobacteriaceae*,* Acidothermaceae*, and *Gemmatimonadaceae.* The relative abundance of *Planctomycetaceae* in the topsoil layer of E treatment was significantly higher than that of F treatment. The relative abundance of *Planctomycetaceae* and *Acidobacteriaceae* in the topsoil layer, of E, F, N, and CK treatment was higher than that of the subsoil samples, while the relative abundance of *Acidothermaceae* and *Gemmatimonadaceae* was similar in the two soil depths. There were 37–60% unclassified tags (Supplementary Table S3).

*Acidothermus* was the prevalent genus in CK, and there was not much of a change in its relative abundance between the two soil depths, while in the subsurface layer’s its relative abundance was greater than the topsoil layer’s in the remaining three treatments. In the F treatment, the relative abundance of *Acidothermus* was considerably greater than in the N treatment in both soil depths. *Pseudomonas* was not much more abundant in the topsoil layer of the E treatment than it was in the other treatments (*p* > 0.05). The subsurface layer of N treatment did not result in a statistically significant (*p* > 0.05) increase in *Pseudomonas* relative abundance over other treatments compared to the topsoil. Unclassified tags were more than 70% (Supplementary Table S3).

### The correlation between grass species and soil microbial richness

**Fig. 5:**
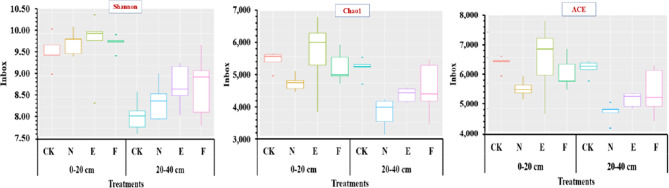
Box diagram of Shannon, Chao1 and ACE for each treatment. CK, N, E, and F: control without grass, natural grass, Reyan No. 2, and Ubon

The richness or evenness of soil microorganisms is expressed by Chao1, ACE, and Shannon. Figure 5 shows how different treatments and soil layers significantly affected the richness and evenness of soil microorganisms. In terms of Shannon index, the microbial richness and evenness of the topsoil layer in CK and N treatments were highly significantly (*p* < 0.01) higher than those in the subsurface soil samples. The microbial richness and evenness in the surface soil samples of E treatment were significantly (*p* < 0.05) higher than those in the subsurface soil samples. The soil microbial richness and evenness were significantly (*p* < 0.01) different among the two soil depths of E, F, and CK (Supplementary Table S4). The findings demonstrated that soil depth was the primary factor influencing the evenness and richness of soil microorganisms.

### Analysis of β- diversity

**Fig. 6:**
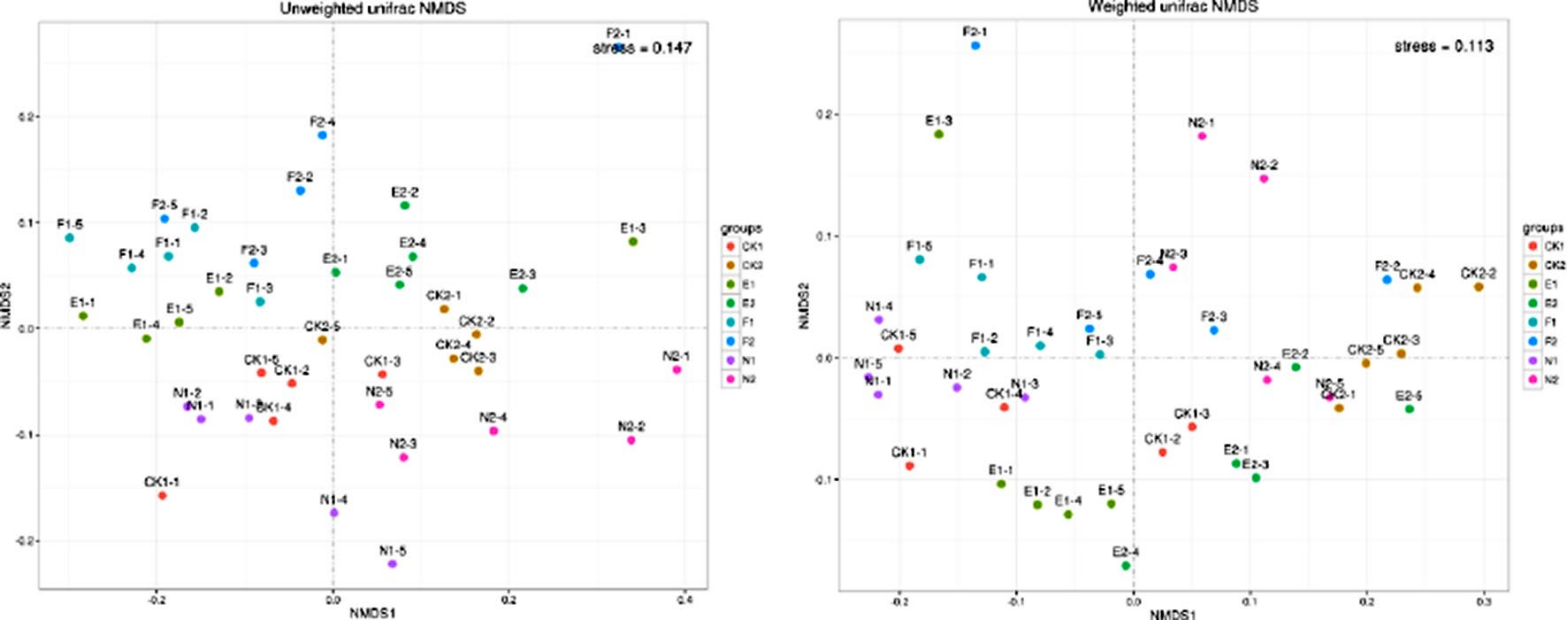
NMDS analysis among different treatments. 1 and 2 refer to top and subsoil sample, respectively. CK, N, E, and F: control without grass, natural grass, Reyan No. 2, and Ubon

The soil microbial β-diversity was computed to investigate the effects of soil depth and grass kinds on the structure and composition of the soil microbiota. β-diversity was investigated by the sample distance calculation method of weighted UniFrac and unweighted UniFrac. UniFrac is a β-diversity metric that compares environmental samples using phylogenetic information. Among them, the unweighted UniFrac only reflects the existence of species, while the weighted UniFrac represents the existence and abundant changes of species. Therefore, the combination of the two UniFrac analysis methods can more effectively find the structural differences between samples. According to the weighted and unweighted UniFrac matrix, NMDS analysis showed that the samples from the same soil depth and grass varieties are concentrated together, as shown in Fig. 6.

**Fig. 7:**
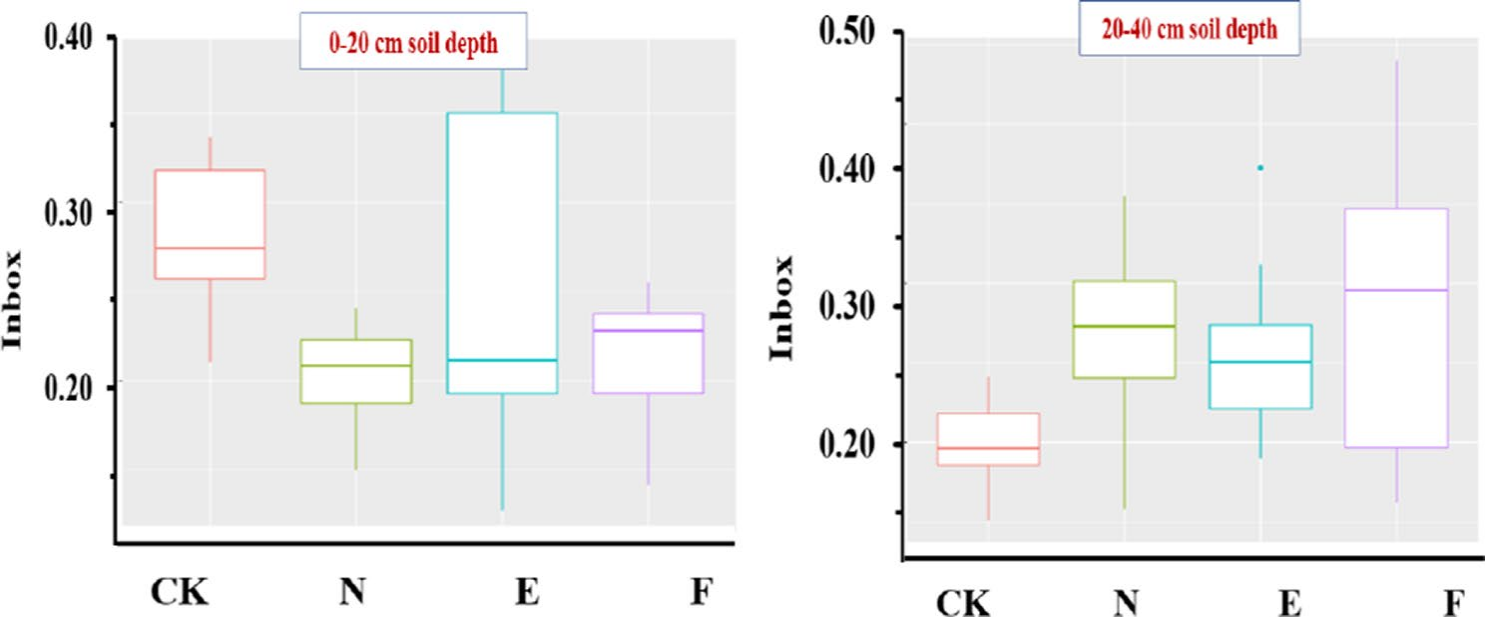
Box plot of UniFrac distance for each treatment. CK, N, E, and F: control without grass, natural grass, Reyan No. 2, and Ubon

Figure 7 and Supplementary Table S5 show that in the surface soil samples, there were highly significant (*p* < 0.01) variations in the soil microbial composition and structure between the CK and F treatments and between N and CK treatments. There was a significant difference among the four treatments of CK, N, F, and E treatments in the topsoil layer (*p* < 0.05). In the subsoil layer, the soil microbial structure and composition between N and CK reached a very significant difference (*p* < 0.01). There was a significant difference (*p* < 0.05) in the quantity and structure of soil microbes between CK and E as well as CK and F. The soil microbial composition reached a significant difference among the four treatments of CK, N, F, and E in the subsurface soil samples (*p* < 0.05). The microbial structure and content of the surface and deep soil samples in the CK and N treatments differed significantly (*p* < 0.01). The soil microbial structure and composition among the two soil depths of the surface and subsurface soil samples of the four treatments, i.e., CK, N, F, and E, were highly significantly different (*p* < 0.01) based on Tukey-HSD analysis (*p* < 0.05) (Supplementary Table S5).

The further Adonis analysis (Supplementary Table S6) showed that in CK and the natural grass treatments, the microbial structure and composition between the two soil depths reached a very significant level (*p* < 0.0 L). In the topsoil layer, the soil microbial composition of F and N decreased significantly compared with CK. The treatments of N, F, and E considerably enhanced the variety of the soil microbial composition in the deep soil layer as compared to CK. The soil microbial composition among the different depths of CK, N, F, and E, reached a very significant level (*p* < 0.01). Compared with CK, N and F treatments significantly reduced the composition of soil microorganisms in the surface soil samples, while E had no significant effect. In the subsurface soil samples, N, F, and E significantly increased the diversity of soil microorganisms. Therefore, the effect of grass cultivation on the composition of the soil microorganisms varied through the soil depths. In the topsoil layer, the effect of grass cultivation, had little effect on the composition of soil microbial community, while grass cultivation had a positive effect on the composition of soil microbial community in the subsoil layer. The soil depth had a greater effect on the composition of soil microbial community compared to the grass species.

### Multivariate analysis

**Fig. 8:**
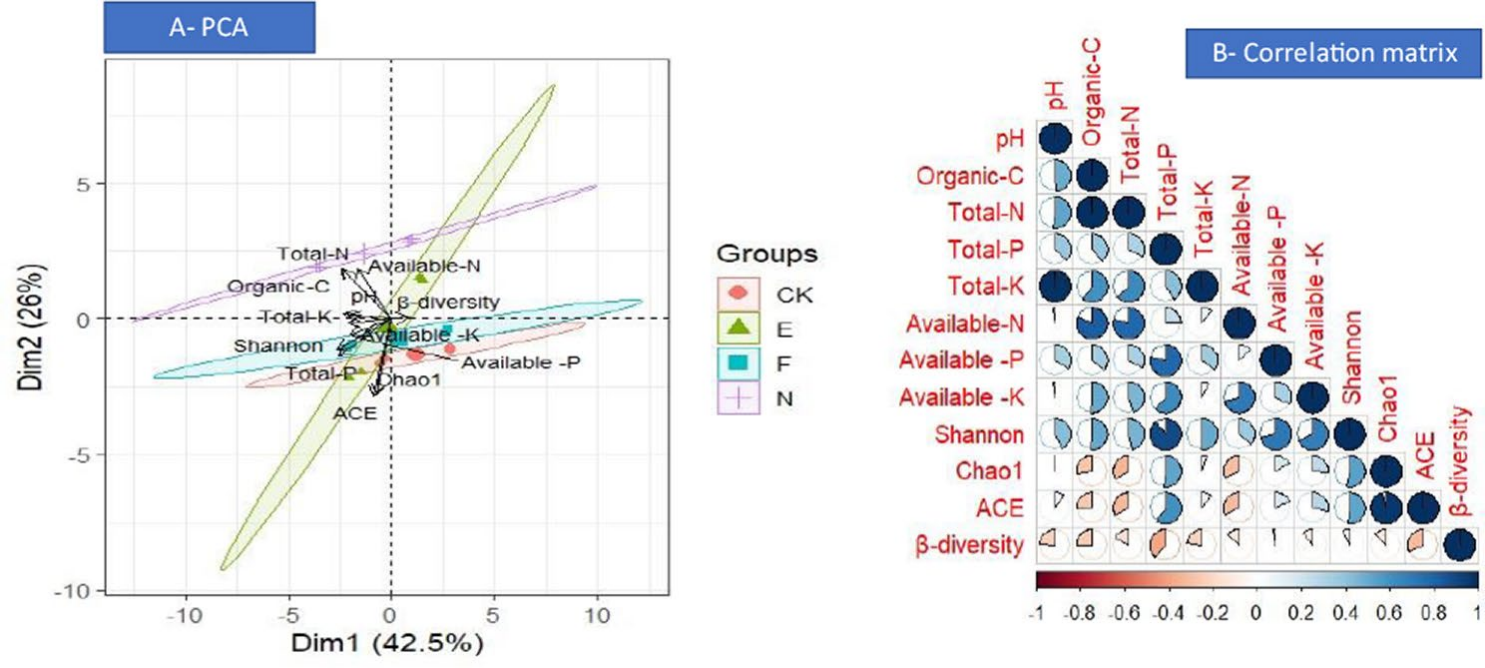
PCA (principal component analysis) (**A**) and the correlation matrix (**B**). CK, N, E, and F: control without grass, natural grass, Reyan No. 2, and Ubon

The intricate relationships between the variety of microbes and the characteristics of the soil were examined using the principal component analysis (PCA) and correlation matrix (Fig. 8). Shannon index was positively and significantly correlated with soil pH, SOC, and soil nutrients, i.e., nitrogen, phosphorus, and potassium. The total soil phosphorus and the Chao1 and ACE indices showed a positive and significant correlation. The PCA findings showed a significant relationship between the soil pH, SOC, and soil N, P, and K, and the Shannon, Chao1, and ACE indices.

## Discussions

In this study, the latosol soil’s overall microbe count and the diversity were altered by the grass-growing technique. Variations in the soil microorganisms were correlated with variations in the soil nitrogen and carbon. Soil microbes are one of the most important components of terrestrial ecosystems and influence the biochemical processes in the soil that govern energy flow and nutrient cycling [14]. Based on the 16 S rDNA analysis technique, the natural grass and Ubon treatments decreased the relative abundance of *Planctomycetaceae* in the surface soil samples, while Reyan treatment improved it. The natural grass and Ubon treatments increased the relative abundance of *Acidobacteriaceae*. The cultivation of Reyan and natural grass enhanced the relative abundance of *Planctomycetaceae* in subsurface soil samples. The natural grass and Ubon treatments increased the relative abundance of *Acidobacteriaceae*, and Ubon increased the relative abundance of *Acidothermaceae*. The relative abundance of *Planctomycetaceae* and *Acidobacteriaceae* in the topsoil layer of Reyan, Ubon, natural grass, and CK were higher than that of the subsoil layer. In line with the findings of this investigation, Jiao [29] also observed that *Acidobacteriaceae* are more abundant in the soil of natural grass, which may be because natural grass has a great variety of plant species [30]. Grass-growing methods significantly increased the number of bacteria, fungi, actinomycetes, and the total number of soil microorganisms [9, 31]. The richness, functional diversity, and rate of use of carbon sources by the soil microbial population might all be enhanced by the growing grass [10, 32, 33].

In the present investigation, the relative abundance of soil microorganisms changed at different classification levels in the tested treatments. *Chloroflexi* was the dominant phylum, with the highest relative abundance, which is inconsistent with other studies of Gupta et al. [34] and Coller et al. [35]. In all of the treatments, *Proteobacteria* was also the predominant bacterial phylum, which is in line with research on the variety of bacteria found in soil [35, 36]. *Acidobacteria* was also one of the most abundant bacteria in soil, next to *Proteobacteria*, which is consisting with the findings of An et al. [37]. The proportion of Proteobacteria and *Acidobacteria* in the soil may serve as a proxy for several environmental factors, including the pH of the soil and the amount of organic and inorganic materials present [1, 10]. *Acidobacteria* grow under oligotrophic conditions, which can diagnose the characteristics of individual species using a variety of carbon substrates and indicate potential activity in the soil [38]. Organic carbon and nutrients also play key roles in the microbial community [39].

The richness and evenness of soil microbes are mostly reflected by the Shannon index. In this study, according to Shannon index, the richness and evenness of soil microorganisms in the topsoil layer of Reyan, Ubon, natural grass, and CK were higher than those of the subsoil layer. The richness and evenness of soil microorganisms in all the grass treatments were significantly higher than those in CK at the two soil depths. The results showed that soil depth had the greatest impact on the evenness and richness of soil microbiology. There are few reports on the effects of land use patterns on soil microorganisms [40]. The long-term land use type change [41] and tillage system [42] have little effect on bacterial richness. The topsoil has a stronger influence on soil microorganisms than the subsurface [43], which is compatible with the findings of this research. In light of the findings of this investigation, it can be stated that the composition of the soil microbial community is closely linked to the soil nutrients, which serve as an important marker of soil quality. Therefore, the grass cultivation changed the soil properties by changing soil nutrient content, resulting in soil microbial evenness and richness depending on soil depth. The main reason is that the grass varieties are shallow root plants, and their roots are mainly concentrated in the upper soil. Therefore, the richness and evenness of soil microorganisms mainly depend on soil depth. Another reason is that the upper soil is easily disturbed by other influences, e.g., pesticides, fertilizers and rainfall, which are also the primary forces behind the alteration of the soil microbial community [44].

β-diversity is a term used to describe how biological organisms respond to changes in their environment, such as shifts in species and generation. Through the use of Adonis and UniFrac distance difference analysis, the study found that natural and Ubon grasses significantly lowered the variety of soil microbial community composition in the topsoil layer, whereas Reyan had no discernible effect when compared to CK. In the subsoil layer, the natural grass treatment significantly augmented the diversity of soil microbial community composition. Depending on the soil depth, grass cultivation has varying impacts on the variety and composition of soil microorganisms. The variety of the soil microbial population in the subsurface layer was markedly enhanced by the natural, Ubon, and Reyan grasses. This might be because the rhizosphere exudates and plant species richness of the natural grass, Reyan, and Ubon treatments add to the variety of the soil microbial community composition. The grass cultivation technique in forest soil could significantly change the soil β-diversity of orchards [33]. Grass intercropping altered the nature of the soil’s microbial community and increased the quantity of carbon, nitrogen, and organic matter in the soil [10, 45]. Furthermore, it fosters an environment that is favorable to the development and production of the bacterial community and raises nutrient bioavailability, all while encouraging bacterial population activity in nutrient cycling. According to a prior study, intercropping grass improved soil aeration and water permeability by increasing the amount of soil organic carbon. Porous soil aggregates were produced as a result of the above-mentioned soil process [10, 46]. Therefore, it might effectively increase the soil’s capacity to retain water in orchards, accelerate the microbial breakdown, increase the quantity of root exudates, activate the mineral components of the soil, and increase the soil organic compounds [1, 47].

Soil health of tropical soil ecosystems is determining by the interactions between soil properties and microbiome. Leguminous and natural plants enhance nitrogen and phosphorus availability in the soil, thereby promoting microbial diversity and activity [48]. The interaction of plant, soil properties, and the microbiome not only improves nitrogen and phosphorus content but also influences soil pH and organic matter composition, creating favorable conditions for beneficial microbial communities [19, 48]. The combined effects of plant-microbe interactions lead to improved soil structure, nutrient cycling, and overall soil fertility, thereby influencing the productivity and sustainability of guava orchards [19, 48].

## Conclusion

The use of tropical legume grasses and the natural grasses on the tropical orchards significantly increased the biodiversity of soil microorganisms based on 16 S rDNA technique. The top soil layer’s soil microbial richness and evenness in the grass treatments were higher than those in the subsoil layer. The soil microbial evenness and richness based on soil depth resulted from the alterations in soil nutrient and organic carbon content caused by the grass cultivation. The majority of the roots of tropical legume grasses and natural grasses are found in the top soil, making them shallow rooting plants compared to the deep roots of orchard trees. The findings offer a theoretical foundation for enhancing soil quality in tropical orchard soils by the growth of grass in latosol orchards; nevertheless, more long-term field experiments are required.

## Electronic supplementary material

Below is the link to the electronic supplementary material.

Supplementary Material 1

## Data Availability

The datasets generated and/or analyzed during the current study are available in the NCBI SRA database at https://www.ncbi.nlm.nih.gov/sra under the project number of PRJNA1212005.

## References

[1] Xiang Y, Chang SX, Shen Y, Chen G, Liu Y, Yao B, Xue J, Li Y. Grass cover increases soil microbial abundance and diversity and extracellular enzyme activities in orchards: A synthesis across China. Appl Soil Ecol. 2023;182:104720. 10.1016/j.apsoil.2022.104720.

[2] Wang P, He R. Short-term effects of cover grass on soil microbial communities in an Apple orchard on the loess plateau. Forests. 2021;12(12):1787. 10.3390/f12121787.

[3] Xiao L, Lai S, Chen M, Long X, Fu X, Yang H. Effects of grass cultivation on soil arbuscular mycorrhizal fungi community in a Tangerine orchard. Rhizosphere. 2022;24:100583. 10.1016/j.rhisph.2022.100583.

[4] Yan-ting W, Xiao-hao JI, Yu-sen WU, Zhi-quan MAO, Yuan-mao J, Fu-tian P, Zhi-qiang W, Xue-sen C. Research progress of cover crop in Chinese orchard. Chin J Appl Ecol. 2015;26:1892–900. 26572047

[5] FAO. 2020. Food and Agriculture Organization of the United Nations. Available online at: http://www.fao.org/faostat/zh/#data/QC (last update 12. 22. 2020).

[6] Zhao C, Gao B, Wang L, Huang W, Xu S, Cui S. Spatial patterns of net greenhouse gas balance and intensity in Chinese orchard system. Sci Total Environ. 2021;779:146250. 10.1016/j.scitotenv.2021.146250. 33744568 10.1016/j.scitotenv.2021.146250

[7] Rumpel C, Amiraslani F, Chenu C, Garcia Cardenas M, Kaonga M, Koutika LS, Ladha J, Madari B, Shirato Y, Smith P, Soudi B, Soussana JF, Whitehead D, Wollenberg E. The 4p1000 initiative: opportunities, limitations and challenges for implementing soil organic carbon sequestration as a sustainable development strategy. Ambio. 2020;49:350–60. 10.1007/s13280-019-01165-2. 30905053 10.1007/s13280-019-01165-2 PMC6889108

[8] Fang LF, Shi XJ, Zhang Y, Yang YH, Zhang XL, Wang XZ, Zhang YT. The effects of ground cover management on fruit yield and quality: a meta-analysis. Arch Agron Soil Sci. 2021;1–13. 10.1080/03650340.2021.1937607.

[9] Lili J, Gong Q, Wu H, Sheng F, Sun R. Effects of different grasses cultivation on Apple orchard soil microbial community. Chin J App Ecol. 2019;30(10):3482–90. 10.13287/j.1001-9332.201910.039 31621235

[10] Li T, Wang Y, Kamran M, Chen X, Tan H, Long M. Effects of grass inter-planting on soil nutrients, enzyme activity, and bacterial community diversity in an Apple orchard. Front Plant Sci. 2022;13:901143. 10.3389/fpls.2022.901143. 35837455 10.3389/fpls.2022.901143 PMC9274827

[11] Zhang M, Li Z, Zhang B, Zhang R, Xing F. Planting grass enhances relations between soil microbes and enzyme activities and restores soil functions in a degraded grassland. Front Microbiol. 2024;15:1290849. 10.3389/fmicb.2024.1290849. 38426067 10.3389/fmicb.2024.1290849 PMC10903263

[12] Vukicevich E, Lowery T, Bowen P, Úrbez-Torres JR, Hart M. Cover crops to increase soil microbial diversity and mitigate decline in perennial agriculture. A review. Agron Sustain Dev. 2016;36:1–14. 10.1007/s13593-016-0385-7.

[13] Tang J, Yin J, Davy AJ, Pan F, Miao R, Han X. Changes in soil fertility and microbial communities following cultivation of native grassland in Horqin sandy land, China: a 60-year chronosequence. Ecol Process. 2023;12(1):18. 10.1186/s13717-023-00431-2.

[14] Wu H, Cui H, Fu C, Li R, Qi F, Liu Z, Yang G, Xiao K, Qiao M. Unveiling the crucial role of soil microorganisms in carbon cycling: A review. Sci Total Environ. 2023;168627. 10.1016/j.scitotenv.2023.168627. 10.1016/j.scitotenv.2023.168627 37977383

[15] Dobrovol’skaya T, Zvyagintsev I, Chernov AV, Golovchenko GM, Zenova LV, Lysak NA, Manucharova OE, Marfenina LM, Polyanskaya AL, Stepanov MM. Umarov the role of microorganisms in the ecological functions of soils. Eurasian Soil Sci. 2015;48:959–67. 10.1134/S1064229315090033.

[16] Li YM, Wang SP, Jiang LL, Zhang LR, Cui SJ, Meng FD, et al. Changes of soil microbial community under different degraded gradients of alpine meadow. Agric Ecosyst Environ. 2016;222:213–22. 10.1016/j.agee.2016.02.020.

[17] Qian X, Gu J, Pan HJ, Zhang KY, Sun W, Wang XJ, et al. Effects of living mulches on the soil nutrient contents, enzyme activities, and bacterial community diversities of Apple orchard soils. Eur J Soil Biol. 2015;70:23–30. 10.1016/j.ejsobi.2015.06.005.

[18] Borowik A, Wyszkowska J, Kucharski J. Impact of various grass species on soil bacteriobiome. Diversity. 2020;12(6):212. 10.3390/d12060212.

[19] Wei C, Qiao J, Ma Z, Ma H, Tang X, Zhao W, Yan Q, Tang L, Du L. Stylo grass affecting soil respiration in latosol guava orchard of Southern subtropical region of China. Agron J. 2020;113:721–33. 10.1002/agj2.20437.

[20] Schultze-Kraft R, Hubiao Y, Jun T, Guodao L. *Stylosanthes Guianensis* CIAT 184–review of a tropical forage legume. Trop Grasslands-Forrajes Tropicales. 2023;11(2):95–120. 10.17138/tgft(11)95-120.

[21] Olivares J, Valencia E, Ramos-Santana R. Seeding rates of Ubon Stylo (*Stylosanthes guianensis*) affect plant population density, growth and dry matter yield. J Agr Univ Puerto Rico. 2015;99(2):135–46. 10.46429/jaupr.v99i2.3029.

[22] Lu RK. Analytical methods for soil and Agro-Chemistry. China Agric. Sci. Technol; 2000.

[23] Magoč T, Salzberg SL. 2011. FLASH: fast length adjustment of short reads to improve genome assemblies. Bioinformatics 27.21 (2011): 2957–2963. 10.1093/bioinformatics/btr507 PMC3198573 21903629

[24] Caporaso JG, Kuczynski J, Stombaugh J, Bittinger K, Bushman FD, Costello EK, Fierer N, Peña AG, Goodrich JK, Gordon JI, Huttley GA. QIIME allows analysis of high-throughput community sequencing data. Nat Methods. 2010;7(5):335–6. 10.1038/nmeth.f.303. 20383131 10.1038/nmeth.f.303 PMC3156573

[25] Bokulich NA, Subramanian S, Faith JJ, Gevers D, Gordon JI, Knight R, Mills DA, Caporaso JG. Quality-filtering vastly improves diversity estimates from illumina amplicon sequencing. Nat Methods. 2013;10(1):57–9. 10.1038/nmeth.2276. 23202435 10.1038/nmeth.2276 PMC3531572

[26] Edgar RC. UPARSE: highly accurate OTU sequences from microbial amplicon reads. Nat Methods. 2013;1010:996–8. 10.1038/nmeth.2604. 10.1038/nmeth.2604 23955772

[27] Pruesse E, Christian Q, Knittel K, Fuchs B, Ludwig W, Peplies J, Glöckner F. SILVA: a comprehensive online resource for quality checked and aligned ribosomal RNA sequence data compatible with ARB. Nucleic Acids Res. 2007;3521:7188–96. 10.1093/nar/gkm864. 10.1093/nar/gkm864 PMC2175337 17947321

[28] Wang Q, Garrity GM, Tiedje JM, Cole JR. Naive bayesian classifier for rapid assignment of rRNA sequences into the new bacterial taxonomy. Appl Environ Microbiol. 2007;73:5261–7. 10.1128/AEM.00062-07. 17586664 10.1128/AEM.00062-07 PMC1950982

[29] Jiao KB. 2014. Study on characteristics of soil microbial community structure and functions for apple orchard by intercropping. PhD Thesis. Shenyang: Shenyang Agricultural University.

[30] Foesel BU, Nägele V, Naether A, Wüst PK, Weinert J, Bonkowski M, Lohaus G, Polle A, Alt F, Oelmann Y, Fischer M. Determinants of *Acidobacteria* activity inferred from the relative abundances of 16S R RNA transcripts in German grassland and forest soils. Environ Microbiol. 2014;16:658–75. 10.1111/1462-2920.12162. 23802854 10.1111/1462-2920.12162

[31] Tianwei X, du Guodong M, Lu D. Soil microbial and soil enzyme activity variation characteristics on sod-culture Apple orchard. North Hortic. 2018;8:85–8.

[32] Futing L, Zhang L, Li X, Li B, Mingyu H, Wang G, X. Effects of inter-row planting grasses on soil organic carbon fractions and soil microbial community of Apple orchard in Weibei dryland. Plant Nutr Fert Sci. 2014;2:355–63.

[33] Pengfei S, Wang W, Li T, Liao Y, Li Y, Wen X. Effects of different mulching measures on soil properties, bacterial community, fruit yield and quality of Luochuan Apple orchard in Shaanxi Province. Acta Hortic Sin. 2019;46(5):817–31.

[34] Gupta S, Kumar M, Kumar J, Ahmad V, Pandey R, Chauhan NS. Systemic analysis of soil Microbiome Deciphers anthropogenic influence on soil ecology and ecosystem functioning. Int J Environ Sci Technol. 2017;14:2229–38. 10.1007/s13762-017-1301-7.

[35] Coller E, Cestaro A, Zanzotti R, Bertoldi D, Pindo M, Larger S, Albanese D, Mescalchin E, Donati C. 2019. Microbiome of vineyard soils is shaped by geography and management. Microbiome 7, 140 (2019). 10.1186/s40168-019-0758-7 10.1186/s40168-019-0758-7 PMC6839268 31699155

[36] Erping C, Fan X, Li Z, Liu Y, Neal L, Hu C, Gao F. Variations in soil and plant-microbiome composition with different quality irrigation waters and Biochar supplementation. Appl Soil Ecol. 2019;2019(142):99–109.

[37] An FQ, Diao Z, Lv JL. Microbial diversity and community structure in agricultural soils suffering from 4 years of Pb contamination. Can J Microbiol. 2018;64:305–16. 10.1139/cjm-2017-0278. 29401407 10.1139/cjm-2017-0278

[38] Philippot L, Andersson GE, Battin TJ, Prosser JI, Schimel JP, Whitman WB, Hallin S. The ecological coherence of high bacterial taxonomic ranks. Nat Rev Microbiol. 2010;8:523–52. 10.1038/nrmicro2367. 20531276 10.1038/nrmicro2367

[39] Van Horn DJ, Okie JG, Buelow HN, Gooseff MN, Barrett JE, Takacs-Vesbach CD. Soil microbial responses to increased moisture and organic resources along a salinity gradient in a Polar desert. Appl Environ Microbiol. 2014;80(10):3034–43. 24610850 10.1128/AEM.03414-13 PMC4018898

[40] Stenuit B, Agathos SN. Deciphering microbial community robustness through synthetic ecology and molecular systems synecology. Curr Opin Biotechnol. 2015;33:305–17. 10.1016/j.copbio.2015.03.012. 25880923 10.1016/j.copbio.2015.03.012

[41] Goss-Souza D, Mendes LW, Borges CD, Baretta D, Tsai SM, Rodrigues J. 2017. Soil microbial community dynamics and assembly under long-term land use change. FEMS Microbiol. Ecol. 2017;93. 10.1093/femsec/fix109 10.1093/femsec/fix109 28961809

[42] Hartman K, van der Heijden M, Wittwer RA, Banerjee S, Walser JC, Schlaeppi K. Cropping practices manipulate abundance patterns of root and soil Microbiome members paving the way to smart farming. Microbiome. 2018;6:14. 10.1186/s40168-017-0389-9. 29338764 10.1186/s40168-017-0389-9 PMC5771023

[43] Eilers K, Debenport S, Anderson S, Fierer N. Digging deeper to find unique microbial communities: the strong effect of depth on the structure of bacterial and archaeal communities in soil. Soil Biol Biochem. 2012;50:58–65. 10.1016/j.soilbio.2012.03.011.

[44] Wakelin S, Macdonald L, Rogers S, Gregga AL. Habitat selective factors influencing the structural composition and functional capacity of microbial communities in agricultural soils. Soil Biol Biochem. 2008;40:803–13. 10.1016/j.soilbio.2007.10.015.

[45] Fu Q, Gu J, Li Y, Qian X, Sun W, Wang X, et al. Analyses of microbial biomass and community diversity in Kiwifruit orchard soils of different planting ages. Acta Ecol Sin. 2015;35:22–8. 10.1016/j. chnaes.2015.04.002.

[46] Linquist BA, Adviento-Borbe MA, Pittelkow CM, van Kessel C, van Groenigen KJ. Fertilizer management practices and greenhouse gas emissions from rice systems: a quantitative review and analysis. Field Crop Res. 2012;135:10–21. 10.1016/j.fcr.2012.06.007.

[47] Hipps NA, Samuelson TJ. Effects of long-term herbicide use, irrigation and nitrogen fertiliser on soil fertility in an Apple orchard. J Sci Food Agric. 1991;55:377–87. 10.1002/jsfa.2740550306.

[48] Abd-Alla MH, Al-Amri SM, El-Enany AWE. Enhancing rhizobium–legume symbiosis and reducing nitrogen fertilizer use are potential options for mitigating climate change. Agriculture. 2023;13(11):2092.

