# Supplementary figures and images for: Comparison between tropical legumes and natural grasses in improving tropical rainforest soil health: a case study in guava (Psidium Guajava L.) orchards
Source: BMC Plant Biol. 2025 Mar 25;25:378. doi: 10.1186/s12870-025-06395-z (PMC11934814; doi:10.1186/s12870-025-06395-z)

## Slide 1
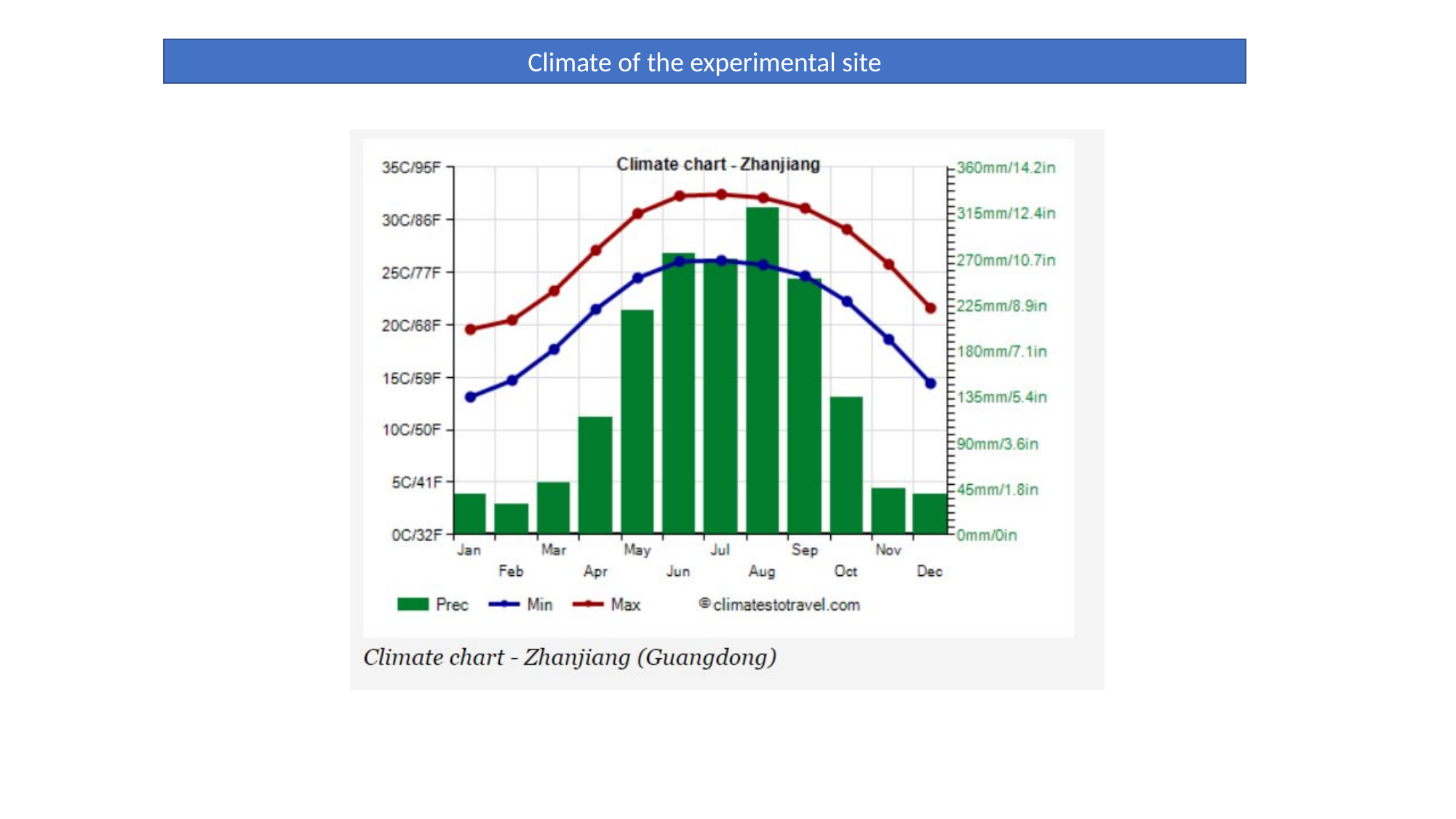

Climate of the experimental site

Supplement: Supplementary file 1 — Supplementary Material 1 [file 12870_2025_6395_MOESM1_ESM.zip › Figure S1.pptx]
